# Surgery: A Practical Treatise with Special Reference to Treatment

**Published:** 1891-12

**Authors:** 


Surgery: A Practical Treatise, with Special Reference to Treatment. By C. W. Mansell Moullin, M. A., M. D., Oxon. Fellow of the Royal College of Surgeons; Surgeon and Lecturer on Physiology to the London Hospital; formerly Ratcliffe Traveling Fellow and Fellow of Pembroke College, Oxford, England. Assisted by various writers on special subjects. With 500 illustrations, 200 of which have been made for this work from special drawings. Octavo, pp. viii.—1180. Philadelphia: P. Blakiston, Son & Co. 1891. Buffalo: Peter Paul & Brother.

The purpose of the author in this volume is to give an epitome of modern surgery, in which theories and disputed opinions are subordinated to practical surgery. The author adopts the somewhat novel but excellent method of describing under the head of each organ the malformations to which it is liable, and the various operations that may be performed upon it, rather than putting such matters in separate chapters. In the author's opinion, "the chief aim and object of surgery at the present day, is to assist the tissues in every possible way in their struggle against disease," and this idea pervades the entire book. He first discusses the general pathology of surgical diseases, including injury and repair, diseases due to non-infective and infective organisms and tumors; then the general pathology of injuries, the general and local effects of injury, burns and scalds, coming under this heading; and, finally, the diseases and injuries of special structures are considered concisely but comprehensively, and this portion makes up the large bulk of the work. The pathology and treatment, as given in the work, are modern, the latter in the main showing a very happy combination of the conservative and the radical. Good practical hints, evidently the result of a large experience in surgery, abound and add much to the value of the book. Some curious omissions occur in a work bearing the date of 1891, such as the non-recognition of the chisel and gouge as a safe and rapid way of entering the skull, and of open arthrotomy in irreducible recent dislocations of some joints, but in the main the book is essentially up to date, and well worth a place in every physician's library.

The illustrations are numerous—some five hundred in all—of which two hundred were made from special drawings for this work. They are excellent for the most part, and illustrate the meaning of the text very clearly. The publishers are to be congratulated on the successful part they have played in giving this valuable work to the profession. J. P.

